# Shaping migration at the border: the entangled rationalities of border control practices

**DOI:** 10.1186/s40878-020-00214-0

**Published:** 2021-02-18

**Authors:** Christin Achermann

**Affiliations:** Centre for Migration Law and Laboratory for the Study of Social Processes, University of Neuchâtel, Rue Abram-Louis Breguet 1, CH-2000 Neuchâtel, Switzerland

**Keywords:** Border-control practices, Organisations, Street-level bureaucrats, Switzerland

## Abstract

This article analyses how border guards as members of a state organisation shape the movement of non-nationals into the territory of a nation state. Based on ethnographic fieldwork on the Swiss Border Guard (SBG), it explores the rationalities—understood as stabilised ways of reasoning and acting—that characterise practices within this state organisation. Combining organisational and structuration theory with a street-level bureaucracy perspective allows for a differentiated analysis of the various facets of border guards’ everyday work. Four rationalities of border-control practices are identified and compared: security, humanitarian, cost-calculation, and pragmatic rationality. I argue that, by considering both the specific goals and imperatives of border control and the characteristics of street-level bureaucrats acting within a state organisation, these entangled logics explain the complex and incoherent social reality of border control. More generally, the results contribute to organisational theory by pointing to the importance of taking into account that multiple entangled rationalities structure the practices of an organisation’s members.

## Introduction

Nation states require that non-citizens meet specific criteria if they are to be allowed entry to their territory. As in other fields of migration control, however, border-control practice never exactly mirrors the law, causing a ‘gap’ and incoherence between policy goals and their implementation (Ellermann 2009; Eule et al. 2019; Hollifield et al. 2014). Going beyond an analysis of policies and laws, this article looks at how border guards control border-crossers and the border itself via concrete, everyday practices (see Côté-Boucher et al. 2014; Lipsky 2010; Loftus 2015; Pallister-Wilkins 2015; Valdez et al. 2017). Its focus is how border guards, as street-level bureaucrats (Lipsky 2010) acting within a state organisation (the Swiss Border Guard [SBG]), put into practice its rules and mission and co-constitute the organisation (Edelman and Suchman 1997; Ortmann et al. 2000). Organisations mediate a broader political and legal context and the everyday practices of those working in their name (Brodkin 2016). Structuration theory (Giddens 1984) helps connect street-level bureaucrat and organisational perspectives by considering that ‘structure’ (the law and organisational resources) and ‘action’ (here, of individual guards) are deeply interwoven and mutually constitutive. Hence, the social reality (Berger and Luckmann 1991) of border control is constituted by the interplay of organisational contexts and ways of doing (Affolter 2021; Alpes and Spire 2014), laws and policies, individual ways of making sense, and everyday actions and interactions.

Consequently, this article seeks to answer the following question: what rationalities structure border guards’ practices when controlling the border and those intending to cross it? Or, put differently, what logics shape how the border is controlled in practice and who can enter a national territory? The analysis identifies four entangled rationalities of border control, which I understand as stabilised ‘ways of thinking and acting’ (Garland 1997, p. 184): security, humanitarian, cost-related, and pragmatic rationality. While the first two are well known in the border-control literature (e.g. Aas and Gundhus 2015; Andreas 2003; Bigo 2014; Pallister-Wilkins 2015; Walters 2011), the others are more generally related to characteristics of organisations and bureaucratic actions. I argue that border guards’ everyday acts that shape the social reality of border regimes cannot be understood solely as a function of the relevant migration laws and border-control policies. Rather, we need to consider and compare multiple rationalities, which originate in structural and organisational norms and conditions and in individual ways of doing and reasoning. Thus, combining street-level bureaucracy and organisational and structuration theory, this article contributes to a differentiated understanding of incoherent and oftentimes illegible migration and border regimes (Eule et al. 2019) by considering how the characteristics of organisations co-constitute border guards’ practices.

Recent analyses of border control have adopted broader perspectives, such as ‘borderscapes’ (C. Brambilla 2015) and ‘border regimes’ (Tsianos and Karakayali 2010). Thereby, they build on scholarship that demonstrates that national borders and their control are not limited to the territorial margins of a nation state but have proliferated to the interior (Ataç and Rosenberger 2019; Pfirter 2019; Yuval-Davis et al. 2018) and the exterior (FitzGerald 2020; Stock et al. 2019) of a state’s territory. Nevertheless, territorial borders remain crucial sites of migration control that perform and reify the differentiation of the world’s population into groups with different rights and privileges (De Genova 2013; FitzGerald 2020). Hence, analyses of border regimes should continue to consider the specific moment of confrontation, at the physical state border, between a non-national and the sovereign power of the nation state.

The next sections situate this article within the border-control practices literature and outline the theoretical framework used. After a methodological account, some contextual information on the legal framework of border control and the SBG as an organisation follows. The main part of the article develops and compares the four identified rationalities, characterising how border guards’ control practices shape migratory movements.

## Border control, a practice perspective

This article situates itself in current scholarship on migration and border regimes (e.g. Eule et al. 2019; Horvath et al. 2017; Pott et al. 2018; Tsianos and Karakayali 2010), which is interested in how policies, laws, and the practices of various actors of unequal power interplay. This literature contributes to an understanding of how nation states govern the movement of people across their borders. My interest in this field is based on the border as a geographical space delimiting a field of action of a state organisation and a certain legal domain (with respect to border-crossing). Building upon critical scholarship (e.g. De Genova 2013; Scheel 2019), I further understand the border and the categories it creates as being reified, performed, and reproduced through the organisation, infrastructure, rules, and practices of border guards. While the law conveys the idea of a clear-cut distinction between whom may enter or not, a close look at the practice side of border guards’ actions reveals additional factors shaping their everyday practices, which thereby constitute the social reality of border control.

Much practice-oriented research on migration bureaucracies follows Lipsky’s (2010) seminal work on street-level bureaucracies. Examining different policy fields and geographical spaces, it bespeaks the variety of practices within these bureaucracies that might result in messy, incoherent processes and demonstrates the important role organisational routines and techniques play in shaping actions and decisions (Affolter et al. 2019; Alpes and Spire 2014; Ellermann 2009; Eule et al. 2019).

Analyses of border-control practices oftentimes concentrate on one particular logic. The most prevalent is a security framing that highlights how security thinking, security personnel, security technology, etc. shape the way border guards act and decide (e.g. Aas and Gundhus 2015; Andreas 2003; Bigo 2014; Côté-Boucher et al. 2014; Loftus 2015). A second rationality is a humanitarian one, taking into account arguments and motivations related to both human rights and human feelings towards border-crossers (e.g. Aas and Gundhus 2015; Fassin 2012; Pallister-Wilkins 2015; Vega 2018; Walters 2011). As demonstrated below, these two rationalities are clearly present in the data analysed for this article. However, I argue that border guards’ actions cannot be reduced to one or two rationalities. To understand why certain people are controlled or not and are granted or refused entry, we need to expand our perspective to take in various entangled rationalities of reasoning and action that result from intertwining legal and organisational structures and individual action. Including an organisational and practice perspective on border control thus improves our understanding of this field.

## Border-control practices, a street-level, organisational perspective

To identify and compare the rationalities structuring control practices at the Swiss border, this article draws on three theoretical approaches: structuration, street-level bureaucracy, and organisational theory. Combining them helps us to consider various shaping factors and to ‘develop a more holistic understanding of border practices’ (Loftus 2015, p. 115).

Giddens’ (1984) structuration theory as an overall framework (Ortmann et al. 2000) highlights the interplay of structural aspects and the actions of, here, border guards, who put legal provisions into practice and thereby shape border control. According to Giddens, structure is formed by rules and resources. This allows one to consider laws and organisational guidelines, but also resources as means of and constraints on action, including time and financial means. In structuration theory terms, border guards are ‘agents’, meaning they are capable, powerful and reflexive subjects with agency. They are not, however, completely autonomous, but interact with a structure. Consequently, the ‘duality of structure’ tells us that structure and (inter)action are inherently related and mutually conditioned. Rules and resources (so, structure) both enable and constrain action. But structures do not exist independently of action; they depend on being realised through action.

Lipsky’s (2010) street-level bureaucracy perspective explains more specifically how front-line state agents within bureaucratic organisations put policies and laws into practice. Lipsky argues that street-level bureaucrats shape the content and orientation of policies while implementing them. Since laws and guidelines are hardly ever completely precise and determined, this perspective draws our attention to street-level bureaucrats’ discretional power, which allows them to choose their own way of proceeding and requires that they make specific decisions. To understand these practices, we need to consider the interpretive schemes, individual interpretations, and priorities they rely on. Additionally, Lipsky highlights the importance of the organisational context in which interactions and decisions take place, in particular working conditions and the means at street-level bureaucrats’ disposal.

Organisational theory helps us focus on these aspects by considering the meso-level of action, which mediates between overarching structures and individuals’ actions. Organisations are groups of people whose conscious, targeted action is aimed at the achievement of specific goals and the implementation of a specific order (Giddens and Birdsall 2004, pp. 346, 348). Building on structuration theory, we see that organisations are realised and reproduced by the fact that their goals, rules and resources are used and referred to in and through the actions of the organisation’s members (Ortmann et al. 2000, p. 317). Accordingly, we should consider how organisational structures orient the actions of organisations’ members through formal rules, specific resources and organisational interpretive schemes that are transmitted through formal training and informal socialisation (Borrelli 2019; Lalonde 2019). However, this perspective further highlights that organisations’ members do not mechanically implement rules or decide in fully rational ways. Rather, it draws attention to how actors use discretion to cope with the structural conditions, ambiguity, and uncertainty of each situation (Lipsky 2010; March 1991; Ortmann et al. 2000).

In sum, combining organisational, street-level bureaucracy, and structuration theory helps us analyse the ‘dialectics of formal organisations and real practices, official regulations and informal norms’ (Bierschenk and Olivier de Sardan 2019, p. 248) and to understand the complex social reality of border control.

## Methodology

The data for the present analysis were collected between December 2015 and February 2017 from members of the SBG and at several border posts in southern and north-eastern Switzerland. They cover both structural elements (e.g., laws, directives, public discourses) and individual border guards’ practices and discourses.^1^ The majority of the ethnographic data was collected at the southern border, in Chiasso Ferrovia, over 19 days of extensive observation at the train station border post and some observations during mobile controls. Observations, countless conversations and informal interviews with many border guards in different functions allowed access to ‘the informal and unexpected’ (Bierschenk and Olivier de Sardan 2019, p. 244). Most of those observed were front-line guards, some of them post leaders, the vast majority white males. These data are completed by two formal interviews with three senior middle- and top-ranking border guards and by written information, including the relevant legal bases and the SBG’s website, publications, and presentations.

The SBG is part of the ‘migration control field’, which is difficult for researchers to access (Kalir et al. 2019). Despite concerns about increasing workload, we were quickly granted an interview with the head of the SBG. An appointed contact subsequently referred us to border guards on the ground and we were able to carry out what is the first ethnographic research at the SBG. Interviews and permission to observe at various sites and times required repeated negotiation and it was difficult to understand why sometimes neither were possible and at others each seemed strictly guided. On occasion, forbidding observing in certain places was justified by citing tactical or security concerns. Whether such restrictions were intended to control our knowledge (Rosset and Achermann 2019) is difficult to assess.

The majority of the fieldwork occurred in the rather exceptional context of summer 2016 (see Häberlein 2019b), which was marked by an important increase in the number of intercepted irregular border-crossers.^2^ The situation at the Italian–Swiss border elicited much public debate throughout 2016. Besides the question of how to respond to the high number of people intending to enter Switzerland, civil society actors criticised how border guards dealt with migrants at the border. The SBG was reproached for illegal pushbacks to Italy (ECRE 2016), for applying excessively restrictive conditions for filing an asylum application (Brambilla and Rozzi 2017), and for using ‘racial profiling’ (Häberlein 2019a). Swiss authorities insisted that they respected the law when refusing thousands entry to the country (Meier 2016). This context put great pressure on the SBG as an organisation and on its individual members, and needs to be considered in our analysis. Its influence should not, however, be overestimated since such moments mostly accentuate pre-existing organisational practices and discourses, reflecting established ways of acting.

## Legal and organisational context

To grasp the structure—understood as constraining and enabling rules and resources—that border guards interact with, some information on the legal and organisational context is needed.

### Legal context

The legal context of Swiss border control is defined by national and supranational law. As a result of Switzerland’s bilateral agreement with the European Union (EU) on the free movement of persons, its membership of the Schengen Area, and the Dublin regulations, the country is bound by supranational rules regarding border control and asylum. These rules forbid systematic identity checks at national borders and govern the responsibility for asylum claims. As Switzerland is not a member of the EU or its Customs Union, national law remains influential. Standard border posts at the national border continue to exist for the purpose of customs control, and the identity of people entering the country may also be checked.

Border control is governed by both migration and customs legislation.^3^ The relevant rules reflect a control and security focus and generally grant the officials responsible vast discretionary power. Security concerns regarding ‘public security and order’ and the ‘internal security of the country and the protection of the population’ feature prominently in both types of legislation and are the dominant provisions that legitimise control of persons and refusal of entry. Dispositions regarding whom and how to control at the border imply vast discretion, and the same is true for rules regarding what is considered an asylum application.

### The SBG as an organisation

The origins of the SBG date back to 1915, when the first federal measures to control immigration at the Swiss border were introduced. This context was marked by war and by worries about protecting the country and its population and resources from foreign nationals who might be a burden or even a danger, but who could not be expelled (Gast 1997). In addition to this general security context, which has framed border control from its inception, the institutions cooperating with the SBG have been part of the security field (military and police). Today, the SBG is a unit of the Federal Customs Administration within the Federal Department of Finances. In 2017, it had around 2000 full-time positions (Graf-Litscher 2017), a number that is regularly debated publicly when the SBG and politicians request it increase (Sicherheitspolitische Kommission NR 2018). The SBG’s internal structure consists of a strategic level at headquarters, an operational level comprising seven border regions, and a tactical level of 38 official border posts that ‘are led by post leaders who ensure the day-to-day performance of their field duties’ (SBG 2020). Since 2002, an SBG ‘migration specialist’ is in charge of ensuring cooperation with the federal migration authorities (Steiner 2017). The SBG stresses its threefold areas of responsibility, which are ‘customs, security and migration’ (SBG 2020). Consequently, the individual border guards on the street-level have to deal with manifold tasks and laws in their everyday work—ranging from controlling the importation of goods to combating money laundering, to fighting against human trafficking or preventing unauthorised entry to or stay in the territory.

## The rationalities of border-control practices

Border guards are in charge of controlling the official border posts and the approximately 150 border-crossing points, but also hundreds of unofficial ‘green border crossings’ and the so-called border zone within the country via mobile controls (Häberlein 2019b, p. 173). Since systematic checks are neither feasible (2.1 million people, 24,000 trucks, and 1.1 million other vehicles cross the Swiss border every day (Eidgenössische Zollverwaltung 2017)), nor allowed in the Schengen area,^4^ border guards must decide where and whom to control. Most of the data this article relies on were collected at the border post at the Chiasso railway station, where trains coming from Italy are stopped and searched before heading north. There, border guards must decide whom to control in a crowded train without causing too much delay or disturbing those making the journey for work or tourism (see Loftus 2015, p. 116). If during a control someone cannot present the necessary authorisation to enter Switzerland, they are taken from the train to the control and registration centre located in the station. There, such people are questioned, their luggage is checked, and a decision is taken to allow entry or to refuse it and return them to Italy.

In what follows, I identify and compare the rationalities that underlie and orient border guards’ control practices. These rationalities reflect the organisation’s properties and mission and simultaneously characterise the organisation and its members’ actions. I understand the notion of ‘rationality’ in a Foucauldian sense, which refers to the fact that ‘particular sets of practices involve, and are organised by, rationalities as specific ways of thinking and styles of reasoning’ (Poertner 2021, p. 51). Rationalities represent stabilised ‘ways of thinking and acting’ and ‘are forged in the business of problem solving and attempting to make things work’ (Garland 1997, p. 184). They do not refer to single actions; nor are they located at the level of the individual actor. Hence, not all of an organisation’s members share all rationalities and rationalities do not have the same importance in each situation. Actors do not necessarily use them consciously or even strategically. Rather, rationalities are situated at the meso-level, where structure and action meet and build a repertoire of ways to reason and act that is constituted by both organisational structure and people’s practices. The following rationalities have been identified through the analysis of the discourses and practices of individuals and of the legal and organisational context.

### Security

The majority of recent scholarship links border control to security (Aas and Gundhus 2015; Andreas 2003; Bigo 2014; Dekkers et al. 2016; Walters 2004). Hence, the security-defining elements of threat, risk, anxieties, control, policing, etc. figure prominently. This reflects the nation state’s perspective on its border, which demarcates the frontier to ‘the outside’ and ‘the other’—both perceived as potentially dangerous. As such, the border is supposed to shield and protect ‘the inside’—the national population, territory, and order (Walters 2004). Studies have demonstrated that those guarding the border also perceive and carry out their job as a security task (Lalonde 2019). Unsurprisingly, security rationality also takes centre stage with the Swiss Border Guard and is at the core of its structure, as represented by the legal provisions regarding border control, how the organisation presents itself, and the SBG’s history, orientation, the professional training it provides, and its proximity to security professionals such as the police and the military. The opening sentences of the SBG’s website—at the time of our fieldwork—highlight the security aspect by presenting the SBG as an ‘armed and uniformed corps’ and as ‘the largest national civil security body in Switzerland’.^5^ The job of border guards is described as being demanding and potentially risky, and training includes shooting exercises (SBG 2020). Interestingly, the SBG explicitly conflates ‘security’ (mostly meaning criminality) and ‘migration’, frequently presenting the two issues as one. For instance, to illustrate ‘security and migration’—which comprise 80% of the SBG’s tasks (20% being customs-related) (Graf-Litscher 2017)—SBG presentations feature the image of a gun and masked individual; its website, meanwhile, describes its task as combating ‘smuggling, irregular migration and cross-border crime’ (SBG 2020). Hence, by fusing migration and criminality, the SBG creates what Bigo (1994) has called a ‘security continuum’, framing migration as a security issue.

At the level of practice, the importance of security is obvious but goes beyond a direct implementation of the legal and organisational structure. Members of the SBG perform their role of ‘guarding’ by wearing uniforms, carrying guns, and displaying a general attitude of suspicion vis-à-vis people who want to enter Swiss territory and the goods they carry with them (see also Häberlein 2019b). Armed with no prior knowledge of those they encounter in a train or vehicle, border guards must quickly assess who might be, for one reason or another (e.g. regarding criminal, customs, or migration law) a threat to Switzerland, its security, and public order and so decide whom to control (Loftus 2015, p. 119). When trains arrive, guards patrol through them quickly, checking toilets for hidden individuals or looking in through windows searching for signs of crime or risk (Jones et al. 2017), never knowing what they might discover (Vega 2018)—guns, drugs, smuggled goods or a lack of requisite travel documents (field notes, summer 2016). According to individual and organisational accounts, border guards rely on observations, ‘risk analysis’, and their ‘cumulated experiences’ to distinguish between suspicious and unsuspicious behaviour and people. According to the authorities, the conditions for controlling a person are ‘clearly defined’, and ‘controls solely based on characteristics such as age, sex, nationality or skin colour are not expedient’ (Meyer 2018). Our own observations (see Häberlein 2019a) and those of other actors (Brambilla and Rozzi 2017; Meyer 2018; Schwarz 2016), however, paint a different picture of control practices in trains. A ‘phenotypic paradigm of suspicion’ (Schwarz 2016, p. 263) resulting in ‘racial profiling’ strongly influences who is asked to present documents or open their luggage.

If a person does not have the requisite documents, suspicion further shapes the ensuing interaction in the registration centre. As a border guard told us, the main question is ‘who this person really is’. In the absence of valid papers proving identity, the agents want to know basic information about the person.

‘We want to know his identity. […]. So, this person is here and claims to be someone. And we want to verify this. And for this reason we need to get information from him: first and last name, date of birth. […]. And very important for us is his biometric fingerprint, […] which allows us to attribute biometry to a person’^6^ (border guard, winter 2017).

Further, guards want to know what individuals want to do in Switzerland—in particular whether they must be allowed to enter because they want to apply for asylum. A frequent way of proceeding, at the time of our observations, was to ask, sometimes repeatedly, if the person wanted simply to go to Switzerland, wanted to work there, or if the person just wanted to cross Switzerland to continue his or her journey, to Germany or elsewhere.

The questioning and counter-checking of people and their biometric data ‘clear’ uncertainty regarding an individual’s identity and intentions (Vega 2018). The concomitant practices of body searches and scrutinising personal belongings reinforce the security character of the encounter. While each border guard has his or her own way of proceeding and communicating, controls often resembled interrogations aimed at detecting risk and unlawful behaviour (see also Häberlein 2019b). Border guards frequently address seated migrants while themselves standing, don’t use the polite form of address (in German or French), ask them to take off their hats or sunglasses, and reprimand allegedly refractory behaviour by shouting. One border guard told us they sometimes have to carry people out of the registration centre if they resist guards’ orders (field notes, summer 2016). These security practices are in sharp contrast with the general public’s expectation regarding how border-crossers who want to seek protection in Switzerland are handled (Häberlein 2019b).

The specific situation of 2016 at the Swiss–Italian border reinforced the guards’ attitude of having to protect the country from ‘masses of people’ seeking entry, which, according to a senior border guard, ‘is really a lot now’. In this context, the sheer numbers and the perceived ‘rush’ of border-crossers created an exceptional context that accentuated pre-existing defensiveness and suspicions.

### Humanitarian

Compared to the security rationality, the second logic works quite differently. Recent scholarship has highlighted the humanitarian character of the border-control practices of different actors at different sites (e.g. Aas and Gundhus 2015; Pallister-Wilkins 2015; Vega 2018). According to Fassin (2012, p. 2), ‘humanitarian’ refers to a dual understanding of humanity. On the one hand, all human beings, based on their shared condition, enjoy universal (human) rights. On the other, ‘humanity’ refers to affection felt for those who are similar, and thus relates to the obligation to assist others. Human rights only manifest marginally in the SBG’s or border guards’ discourses, mostly in relation to being an obvious part of their professionalism and doing a good job. This contrasts with Frontex, whose discourse has lent human rights greater importance recently (Aas and Gundhus 2015; Pallister-Wilkins 2015).

The second characteristic of ‘humanitarian’, related to border guards’ emotions and sentiments of compassion and alleviating suffering, is present in how certain guards talk about their job. Eggebø (2013, p. 313) discusses the ‘fundamentally ambiguous status’ of emotions in bureaucratic work. Emotions can be positive and useful but also burdensome and problematic. One guard mainly referred to the difficult dimension of compassion and emotions he has to deal with in his job: ‘It’s always these individual fates that make you think’. And, ‘At a certain point you have to stop. I mean, maybe it’s an illusion to think you should save them. That’s devastating’ (border guard, winter 2017). Just like the immigration bureaucrats Eggebø (2013) analyses, this guard tries to cope with the emotional burden of his job by maintaining a distance and sticking to the rules and his professional duty:

‘Well, if he’s [the migrant] coming for the first time and was really hoping to work here, then I’m sorry. It’s not nice, but I can’t change it. I don’t have another option, do I? This is how Schengen works. He goes back to where he came from’ (border guard, winter 2017).

Not everyone succeeds in this endeavour though, and there are repeated accounts of psychological problems and burnout due to the general working conditions and the emotionally demanding tasks of migration control. ‘It’s depressing if you have to send people back’, one guard repeated several times (field notes, spring 2016).

As these examples demonstrate, the humanitarian rationality is particularly strong when guards relate to refusing people entry to Swiss territory. It is, however, not limited to such negative decisions and also structures the way some individual border guards interact with migrants during control and registration. Like other officials applying exclusionary policies with regard to unwanted individuals, certain border guards interact with migrants by displaying ‘caring control’ (Vega 2018) or ‘compassionate repression’ (Fassin 2012, p. 135)—thus combining the seemingly contradictory actions of policing, controlling, and rejecting with care, sympathy, and compassion towards the ‘vulnerable’. We repeatedly observed how certain border guards showed—through mostly small gestures—sympathy or even compassion towards those controlled, thereby deviating from the generalised security rationality. For instance, they offer water or food or make jokes with children or give them a pen to play with. One guard was obviously worried about a young man who had previously run across the tracks, and asked him empathically, ‘Why did you run? You can die when you do that; you know you shouldn’t do that’ (field notes, summer 2016). Others take time to listen to personal stories or make a phone call to help the person (field notes, summer 2017).

One guard had been deeply touched by an experience with a woman he had to send back to Italy with her children:

‘I was certainly very concerned by that […] woman with her four or five children, the one who didn’t know how to read and write. And her children. I was really deeply moved by that. All children born in a refugee camp […] And I had to send that woman back because she can’t go to Germany like that. That really hurt me. […] I’ve been worried by that for a long time. It really wasn’t easy to send her back’ (border guard, winter 2017).

He tried to help the woman procure the information that would allow her to comply with the legal requirements necessary to transit Switzerland. Unsuccessful, he had to cope with the fact that following the rules meant acting against his personal feelings of compassion.

This example reveals another dynamic inherent in the humanitarian rationality. Humanitarian gestures are more than signs of altruism. They express ways of coping with repressive tasks, to ‘reconcile the moral ambiguity of their job’ (Vega 2018, p. 2546) and ‘establish their own morality through compassionate discourses’ (p. 2544). Kalir specifies that ‘expressions of compassion do not mitigate an otherwise repressive bureaucratic work’ (Kalir 2019, p. 3). Rather, the ‘emotional comfort zone’ compassion creates allows state officials to foster ‘the self-image of civil servants as humane and sensitive actors’ (p. 1). Importantly, this ‘repressive compassion’ does not affect how officials apply the law, but allows them ‘to move ahead with effectively implementing controversial state policies’ (p. 3). For Swiss border guards, the humanitarian rationality also seems to affect less *what* they do and decide than *how* some do so (see also Vega 2018, p. 2554).

### Cost calculation

A look at the everyday practices of border guards reveals a further logic shaping what they do, how they do it, and with what result. Lipsky (2010, p. 29) shows that street-level bureaucrats act ‘under conditions of limited time and information’. Analysing border guards’ practices from this perspective reveals a cost-calculation rationality resulting from the structural conditions of the organisation, especially the resources (staff and time) allocated. While demand for more border guards is long-standing, it was particularly pressing during our fieldwork (Graf-Litscher 2017).

This rationality includes a cost–benefit and efficiency reasoning that structures how border guards put legal provisions into practice. They are aware that they cannot ensure full control of the border: ‘Whenever politicians tell you that the border is safe, this is nonsense. It is only controlled sporadically since there aren’t enough staff’ (field notes, spring 2016). Hence, guards need to decide where to carry out mobile checks in the border area. For this, border guards run a ‘risk analysis’ based on a cost–benefit calculation of their chances of finding people violating the law. ‘They would find migrants in the […] Valley too, one guard says. But if they observe there for one week and catch only ten migrants, it simply wouldn’t be worth it’ (field notes, winter 2017). While general strategic decisions, such as where to carry out operations and what takes priority, are made by the organisation’s hierarchy, more specific tactical decisions are made by the border post leader (Steiner 2017). Individual street-level units, meanwhile, decide on the spot when to go to which of the designated areas.

As mentioned earlier, both for mobile controls and those at border posts guards must decide whom out of all those in a given train or at a specific place to control. Lipsky (2010, p. xiv) explains how, in a context of heavy workload and limited time, street-level bureaucrats develop different techniques, which can imply ‘modes of mass processing’ or ‘stereotyping’. As stated previously, observations and accusations of border guards using ‘racial profiling’ are frequent (Häberlein 2019a). While at the organisational level such actions are overtly declared unacceptable, in informal settings certain border guards justify focusing controls on people of colour as efficient. Thus, in their accounts, this is not racist—it is a rational strategy, increasing the chances of detecting unauthorised border-crossers.

Efficiency, meaning handling people as quickly as possible, also characterised the way identity checks and registration took place at the registration centre during our observations. Certain guards carry out questioning—which also helps them decide whether a person is seeking protection—and body and luggage searches at high speed.

‘“Why Swiss; why Switzerland?” the brisk guy asks again and within 20 seconds the individual is wearing a blue bracelet [indicating the decision taken]. […] Everything really quick. The post leader is watching and lets him continue. The brisk guy handles speedily. It’s unnecessary because he has finished with the fourteen people before several colleagues have searched the few bags they have with them. […] Fourteen people were questioned, searched, their belongings searched and their fingerprints taken; everything within […] 28 minutes’ (field notes, summer 2016).

One border guard confirms that

‘The process of clearance and registration is actually designed to be as quick and smooth as possible. Well, from our side, it’s of course not the idea to ask, “How are you? Would you like to talk about something?”’ (border guard, winter 2017).

Nevertheless, some migrants might tell border guards what they have experienced before arriving at the Swiss border. In contrast to the humanitarian rationality, which would imply listening to the person, the border guard just quoted highlights the cost-calculation rationality, which restricts the probability of personal and potentially compassionate interactions. Limited resources was also the main reason given for why no interpreters assisted the guards, despite a consensus that communication with migrants was problematic^7^: Interpreters cost a lot, take more time, and increase the chances of people starting to tell their stories, which again takes time (field notes, summer 2016).

### Pragmatic

Border guards act in a context that is characterised by many limitations and much uncertainty, which they must handle while fulfilling their legal and organisational missions. Street-level and organisational literature informs us that limited time, inadequate resources, and lack of information are typical of the challenges street-level bureaucrats face (Lipsky 2010; March 1991; Maynard-Moody and Musheno 2012). Brodkin explains that street-level bureaucrats ‘do not do just what they want [...]. They do what they can’ (Brodkin 1997, p. 24). Compared to the three other logics, pragmatic rationality refers to what border guards ‘can do’—within the structural conditions present and in a situation that requires immediate action—with the means at their disposal. This often involves ‘nonchalance’ (Eule 2018) and ‘pragmatic improvisations [as] an expression of their agency within the context of the rules, practices, and roles’ (Maynard-Moody and Musheno 2012, p. S20). One guard, talking about how they get the necessary information from migrants without having a shared language, frames this situation in words that are common in the literature:

‘Well… you get quite creative. But… we don’t have a choice. Interpreters aren’t provided on the spot. […] Basically, you have to accept and take whatever they’re ready to tell you, or able to tell you, about themselves’ (border guard, winter 2017).

Border guards have to pragmatically deal with several challenges: the limitations imposed by the cost-calculation rationality (e.g. no interpreters, efficiency and time pressure, presence of enough staff in the late evening, or information regarding limited place numbers in asylum reception centres), uncertainty regarding the information an individual provides (their identity, migratory intentions, etc.) and the adequacy—according to the law—of decisions to be taken. As the presentation of a border guard’s job on the organisation’s website demonstrates, the SBG is aware of the importance of pragmatic action: ‘Daily work and interactions with a wide range of people create new demands for flexibility on a daily basis. Checking people means evaluating, deciding, and reacting accordingly. This requires a high degree of expertise and insight into human nature’ (SBG 2020). At other times, pragmatic adaptation is needed, when border guards have to wait for a train to arrive and face boredom and uncertainty regarding what might happen next.

Maynard-Moody and Musheno (2012, p. S19) attribute the need to pragmatically improvise to a ‘mismatch between rules and problems’. But the law itself, as a form of general rule, requires pragmatic improvisation in a given situation. Intuition and experience—in addition to having been sensitised to specific situations during training—are repeatedly cited as being crucial to detecting potential unlawful behaviour and recognising possible risk. ‘Responding to my question regarding their way of controlling, one border guard says: “We feel a lot. It’s with [sic] our instinct”’ (field notes, winter 2017). While ‘gut feeling’ and ‘intuition’ are indispensable resources for street-level bureaucrats (Eggebø 2013, p. 307), they might also result in unequal or discriminatory actions.

Another dimension of pragmatic rationality relates to the guards’ awareness of their limited impact on effectively controlling and stopping irregular migration (Eule 2018). However, they pragmatically accept this as a fact and continue doing what is feasible in the given setting. One guard used the metaphor of the gearwheel to illustrate the limitations of individual impact and the importance of the organisation as a whole:

‘And to some extent it’s the situation and you can’t change it [individual fate]. […] We’re just a cog on a gearwheel, which does its job. And the cogs just need to interlock in the best possible way, and you can’t do more’ (border guard, winter 2017).

Consequently, the resulting decision to grant or refuse an individual entry is shaped not only by security, humanitarian, or cost-calculation reasoning but also by pragmatic, ad hoc decisions. Taken together, these rationalities might result in incoherent practices and gaps between policies and their implementation.

## Entangled rationalities

The four aforementioned rationalities are entangled. They interplay in how they shape the structure of border control and the border guards’ everyday actions. As such, they demonstrate that an organisation’s rules and resources and its members’ practices are interconnected. Each rationality’s impact on a concrete action cannot be definitely determined. Rather, their significance differs according to the individual guard, the specific context at certain border posts, and the given situation. Comparing these rationalities nevertheless reveals some general tendencies regarding their relevance and the dynamics between them. The *security* rationality is clearly dominant in structuring border control. It is the main organisational rationale in terms of structure, mission, history, and ‘institutional habitus’ (Affolter 2021) and is rooted in the relevant legal framework. Hence, the SBG and its members pursue what Walters (2004) terms ‘domopolitics’—protecting the national ‘home’ from menacing outsiders by filtering wanted from unwanted mobility. The prevalence of the security rationality at the structural level favours individual guards focusing on suspicion-driven policing to detect and exclude perceived threats. The *cost-calculation* rationality, in turn, is not a result of the SBG’s primary policy goals, but is related to its characteristics as a street-level organisation dealing with limited resources and efficiency pressure. The *humanitarian* rationality manifests mostly in individual border guards’ emotions and ways of interacting with migrants at the border. The *pragmatic* rationality reflects how individual guards deal with the other three, partly conflicting rationalities in specific situations of border control.

The four rationalities can vary in their interaction and impact border guards’ decisions and actions in different ways. Cost-calculation, pragmatic, and humanitarian dimensions can exacerbate or attenuate the dominant security logic. For instance, organisational efficiency reasoning combined with a general suspicion of racialised ‘others’ impacts guards’ ‘gut feeling’ and increases the tendency to control people of colour. The humanitarian rationality is in tension with the security and the cost-calculation rationality, each of which reduces the chances of a border guard investing in personal encounters. Security and humanitarian rationalities, meanwhile, can conflate, resulting in a ‘care and control duality’ (Pallister-Wilkins 2015, p. 54) of caring for migrants while simultaneously controlling their mobility. The pragmatic rationality, while mediating the three other rationalities, risks curtailing the security mission and humanitarian intentions when it relies on improvisation or intuition. Ultimately, the structural conditions of the specific task of border control combined with the organisational imperatives of bureaucratic work—reflected in the cost-calculation and the pragmatic rationality—result in the security rationality prevailing, while restricting the scope of the humanitarian rationality.

Overall, contrary to clear-cut policies and laws distinguishing unambiguously between those to be granted or refused access, practices at the border reveal a more differentiated picture of the border-control regime. The four entangled rationalities demonstrate that the contradictions of migration control not only result from competing logics between different state agencies but also characterise practices within a specific organisation (Eule et al. 2019). The properties of organisations contribute to this contingency by adding constraining or enabling conditions regarding action. Adopting a differentiated and practice-oriented perspective reveals the social reality of border control and the inherent logic of structure and action of this specific field. Thus, considering the characteristics of organisations adds to the literature that seeks to explain the gap between policies and their implementation.

## Conclusion

This article has analysed border-control practices and identified the rationalities that shape how the border is controlled and how decisions to grant or refuse entry to Switzerland come about. The combination of structuration theory with a street-level understanding of organisations drew attention to the interrelated and mutually constitutive character of structure and action. Consequently, the article analysed how structural components, such as legal ‘rules’ and human and organisational resources, constrain and enable individual (inter)actions and co-constitute the organisation. It simultaneously detailed how the legal and organisational conditions regarding border control become socially relevant by being put into practice by border guards—a person only becomes an ‘unauthorised border-crosser’ once categorised as such by a state agent.

The organisational perspective highlights that the SBG mediates between a broader structure and individual actions. I argue that the approach adopted here provides a differentiated understanding of the social reality of border control. Analyses of border-control practices are mostly confined to their specific legal and policy field, thus focusing on security and humanitarian rationalities. This analysis has confirmed that security thinking is paramount in shaping border guards’ reasoning and everyday actions, and that humanitarian reasoning also influences these actions. Adopting an organisational perspective and considering the organisational and situational context of border guards doing their job, meanwhile, has revealed that these logics are intertwined with further rationalities. Thus, whether a non-citizen succeeds in accessing Swiss territory at a border post depends also on a cost-calculation and a pragmatic rationality, which impact how border guards reason and act when putting their legal and organisational mission of controlling the national border into practice. I argue that such a nuanced perspective on the entangled rationalities inherent in both the field of border control and in bureaucratic practices explains some of the gaps and incoherence in border regimes.

More generally, this article demonstrates the importance and potential of analysing migration- and mobility-related processes as general social processes (see Dahinden 2016), in this case by using organisational, street-level bureaucracy, and structuration theory. Conceptualising border guards as street-level bureaucrats acting within a state organisation revealed rationalities rarely mentioned in the border-control literature but that are crucial to an understanding of how they act. Hence, comparing border guards and the SBG with other organisations has helped to advance our understanding of how border-control practices shape migration. At the same time, the insight that entangled rationalities structure the practices of an organisation’s members has the potential to stimulate differentiated analyses of other policy fields.

Finally, to achieve an even more nuanced understanding of the border regime, it would be interesting to include the perspective of border-crossers and the rationalities of their actions and their ways of appropriating the exclusionary policies and state practices (Scheel 2019) resulting from entangled rationalities.

## Data Availability

The data used for this manuscript is subject to privacy regulations and not available to the public.

## Footnotes

1 Fieldwork was carried out by Jana Häberlein.

2 With a total of 48,048 registered in 2016, unlawful entries had more than tripled compared to 2014 (14,265) (Eidgenössische Zollverwaltung 2017, p. 24).

3 The most important laws and articles being The Federal Foreign Nationals and Integration Act (Article 5), the Federal Asylum Act (Article 18) and the Federal Customs Act (Article 100) (see https://www.admin.ch/gov/en/start/federal-law/classified-compilation.html).

4 The exceptional situation due to the COVID-19 pandemic in spring 2020 demonstrated the effects of strictly controlling the border. See https://www.ezv.admin.ch/ezv/de/home/aktuell/medieninformationen/medienmitteilungen.msg-id-79666.html.

5 The site’s present version presents the SBG as ‘an integral and recognised part of the [sic] Switzerland’s security landscape’ (SBG 2020).

6 Most quotations from interviews and field notes were translated from German.

7 After the tensions of 2016 and after our fieldwork was finished, the SBG announced several adaptations to its processes, especially regarding communication (Wirth 2017).
